# A Bengali news and public opinion dataset from YouTube

**DOI:** 10.1016/j.dib.2023.109938

**Published:** 2023-12-10

**Authors:** Lomat Haider Chowdhury, Salekul Islam, Swakkhar Shatabda

**Affiliations:** a Department of Computer Science and Engineering, Ahsanullah University of Science and Technology, Bangladesh; b Department of Computer Science and Engineering, United International University, Bangladesh

**Keywords:** Bengali news,, News media, YouTube, Comments, Public sentiment

## Abstract

Along with the traditional news publishing policies, news agencies now share news over the internet since people nowadays prefer reading news online. Moreover, news media maintain YouTube channels to publish visual stories. Readers comment to share their opinions below the corresponding news item. These news and comments have been a great source of information and research. However, there is a lack of research in the Bengali news context. This article presents a dataset containing 7,62,678 public comments and replies from 16,016 video news published from 2017 to 2023 from a renowned Bengali news YouTube channel. The data withholds 15 properties of news that include video URL, title, likes, views, date of publishing, hashtags, description, comment author, comment time, comment, likes in the comment, reply author, reply time, reply, and likes in the responses. To ensure privacy, the commentator's name is encoded in the dataset. The dataset is open to use for researchers at https://data.mendeley.com/datasets/3c3j3bkxvn/4. A translated file for the raw dataset is also included. This data may help scholars to identify patterns in public opinion and analyze how public opinion changes over time.

Specifications Table

**Table:** 

Subject	Data Science
Specific subject area	Analysis of public opinion in news articles to examine their viewpoints
Data format	Raw, Cleared
Type of data	Table (Excel)
Data collection	Data is collected from a Bengali YouTube channel. Both sequential and keyword-based searching are used for data collection purposes.
Data source location	Institution: United International University (UIU) City/Region: Dhaka Country: Bangladesh The data was collected from an online platform YouTube using the following link: https://www.youtube.com/@somoynews360/videos
Data accessibility	Repository name: Mendeley Data Data identification number: 10.17632/3c3j3bkxvn.4 Direct URL to data: https://data.mendeley.com/datasets/3c3j3bkxvn/4

## 1 Value of the Data

• The data can assist the government and policymakers in perceiving public viewpoints toward national and international events (crises and developments). Moreover, the dataset covers a wide range of topics from politics to economics. So, can be used in surveillance and early detection against any kind of violence.
• The dataset will assist the researchers interested in Natural Language Processing (NLP) to identify public sentiment on news articles. New language models for the Bengali language can be proposed using a large amount of data.
• The dataset contains a total of 7,62,678 public comments from 16,016 distinct news articles data with all necessary information corresponding to the news from YouTube. The researchers can shortlist them on any particular topic for forthcoming research projects. The data can be used in detecting forgery in news and events which can assist to stop the spreading of misinformation.
• The dataset can be used to depict how social media and news media are promoting events. It can determine what events and news are getting more public attention. News agencies will be able to understand how people are responding to their news.
• It can help news channels open new discussion that supports the public interest.
• The dataset can be used for the empirical study to identify cross-cultural differences in Bengali news compared to news article data in other languages. It can also examine the properties of information media sources in Bangladesh compared to other countries.

## 2 Data Description

The data is retrieved from ‘Somoy TV’, a renowned Bengali YouTube news channel with over 20 million subscribers [1]. YouTube is used as a platform for data collection for research purposes by other researchers as well. In [2], authors proposed a dataset for Malayalam movie sentiment analysis from the reviews shared on YouTube. Another dataset is proposed for terrorism-related discussion on YouTube by Kayode-Adedeji et. al [3]. In this article, the proposed dataset contains 7,62,678 comments for 16,016 distinct news videos. It covers diverse news topics, such as politics, local, national, and international news, the economy, entertainment, sports, and education, among many others. The diversity of the data is ensured through keyword-based data collection processes. The raw version of data contained eight features for each news. Later, the comments, replies, and their properties were separated. The dataset contains news from June 21, 2017, to May 23, 2023. The dataset is shared for further research in an Excel file named **banglaNewsData.xlsx**. As mentioned, the dataset is a Bengali dataset. With the advancement of natural language processing, some other research has been conducted on Bengali datasets for NLP purposes [4].

In article [5], authors discussed the properties of big data in the context of sentiment analysis and proposed a cloud-based sentiment analysis approach using Hadoop and Apache Spark for big data. Varied datasets are available in Bengali and other languages for sentiment analysis purposes. An elementary comparison of these with the proposed dataset is depicted in Table 1. The comparison demonstrates that the proposed one outperformed the others in terms of data volume, categories, and attributes.

**Table 1:** A comparison among other relevant datasets.

Specification	KanCMD [6]	Russia-Ukraine War [7]	ABSA [8]	BAN-ABSA [9]	Proposed Dataset [10]
Number of Comments	7,671	10,861	5,091	9,009	7,62,678
Platform	YouTube	YouTube	Facebook, News Portals	News Portals	YouTube
Language	Kannada, Latin, and English	Bengali	Bengali	Bengali	Bengali English (Translated)
Categories of Data	Movie trailers, Trend of banned mobile apps in India, India-China Border, Mahabharat, Transgender	Russia and Ukraine War	Cricket and Restaurant Review	Politics, Sports, Religion, and Others	Latest news, Special news, Politics, Bangladesh, World, Crime, Trade, Opinion, Sports, Entertainment, Job, Lifestyle, Education, Election, FIFA, Earthquake, Price hike, Padma bridge, Metro rail
Attributes or Features	Comments, Class	Video URL, Comments, Annotation	Cricket: Source, Date, Text, Category, Polarity Restaurant: Text, Category, Polarity	Comment, Aspect, Polarity	Video URL, Title, Likes, Views, Publish Date, Hashtags, Description, Commentator, Comment, Comment Time, Comment Likes, Reply Author, Reply, Reply Time, Reply likes

**Fig. 1:**
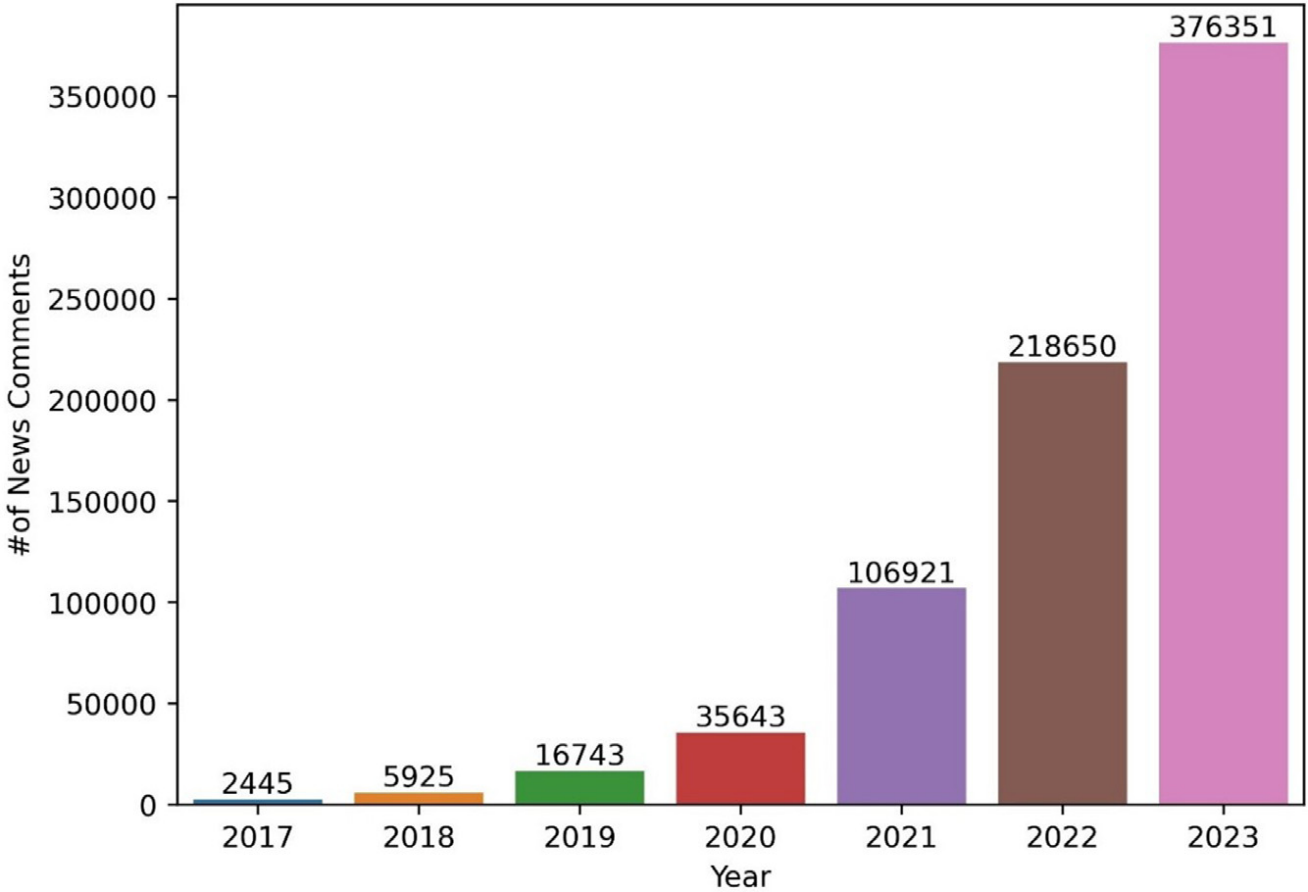
Number of news comments in the dataset from 2017 - 2023.

Fig. 1 represents the number of comments from the year 2017 to 2023. The number of comments shows that viewers are becoming more engaged with YouTube news reports over time.

## 3 Experimental Design, Materials and Methods

Three steps are used to prepare the dataset: data collection, data preparation, and exploratory analysis. The processes are briefly explained in this section.

### 3.1 Data collection

It is necessary to have a suitable dataset covering a variety of news topics to comprehend how native Bengali-speaking individuals feel about national and international news articles. With that goal, the data collection process was initiated. The data is gathered from a particular news article source on YouTube using NodeJS. The Chrome browser is controlled for URL retrieval using the Puppeteer framework.

Both sequential and keyword-based searching are used for data collection purposes. The data collection process was initiated on May 23, 2023. A sequential data collection method is used to retrieve all of the news information from 23 May 2023 back to 27 December 2022. Additionally, a keyword-based search was integrated. A total of 19 keywords are used to retrieve the data. The keywords are mentioned in Table 1 as **Categories of the Data**. The raw data is stored in a JSON file. A total of 22,757 news articles were collected. No duplicate news appearances were checked during the data collection stage.

### 3.2 Features of data

While collecting data, a total of 15 features for each sample were targeted. At first, the URL for the corresponding news data is stored as a feature “videoUrl”. The title for the news articles is the second feature stored as “title”. For most of the samples, the video title is represented as the title in Bengali, the title in English, and the channel name, separated by a vertical bar (|). After that, the total number of likes and the total number of views are stored as two different features named “totallikes” and “views” respectively. Some updates were required for these features that are described in “*Data preparation*”. The date on which the news was published is stored in the “date” feature. The date was stored as a string which was later converted as a Date type using the Pandas *to_datetime()* method. While publishing news, the publishers use some hashtags relevant to the news to make the search convenient to the users. These hashtags were also collected as a feature. Publishers can choose to publish news without hashtags. When no hashtags are found for the news an empty square bracket []. There is a description box below each video on YouTube that involves some relevant information regarding the news or publishers along with Copyright details as well. Discarding the copyrights, the rest of the details are stored in the “description” feature. After that, the comments and replies were collected which are the main focus of this dataset. For each comment and reply, four different attributes were stored as “commentAuthor”, “commentTime”, “comment”, “commentLikes”, “replyAuthor”, “replyTime”, “reply”, and “replyLikes”. Here, the author is the person who comments or replies. To ensure the privacy of the commentators, the name of every person who comments and replies is replaced with a unique author count. “commentTime” and “replyTime” represent the time of the statement made from the data collection date. “comment” and “reply” here are the statement made by people regarding the video which usually depicts public's sentiment and point of views. The number of likes in the statements is stored as “commentLikes” and “replyLikes”. Though there is a dislike option attached to each video, comments, and replies, the value of dislikes couldn't be retrieved. According to YouTube policies of 2021, the count of dislikes will not be public anymore.

### 3.3 Data preparation

When preparing the data, all duplicate news stories are first removed. The elimination process is maintained as such so that the data collected later remains since it may reach more views than the previously collected one. Videos on YouTube that receive more than 1,000 views and 100,000 views are sometimes annotated with the Greek letters K and M, respectively. A similar concept applies to video likes as well. To convert the object type data to numeric form, the Greek annotations are replaced by numbers.

After that, the data is sorted based on their publishing date in ascending order. While collecting the data, all the comments, replies, and respective information were stored as a single attribute. At this point, each comment and reply are separated along with the author's information, time, and likes. To assure the privacy of the author, their names are replaced with annotated values for publicly available datasets. Data is then stored in an Excel file for further implementation.

The dataset is a Bengali dataset which is the novelty of this work. However, an English translated version is also published for research purposes. To translate the Bengali titles, descriptions, comments, and replies, Google Sheets are used as a tool. From Google Sheets, we used the formula of “GOOGLETRANSLATE” which takes three parameters to translate a given context. The first one is the text that we want to translate, the second parameter is the source language for the text, and the third parameter is the target language. For the proposed dataset, the source language was Bengali (bn) and the target language was English (en).

### 3.4 Exploratory analysis

**Fig. 2:**
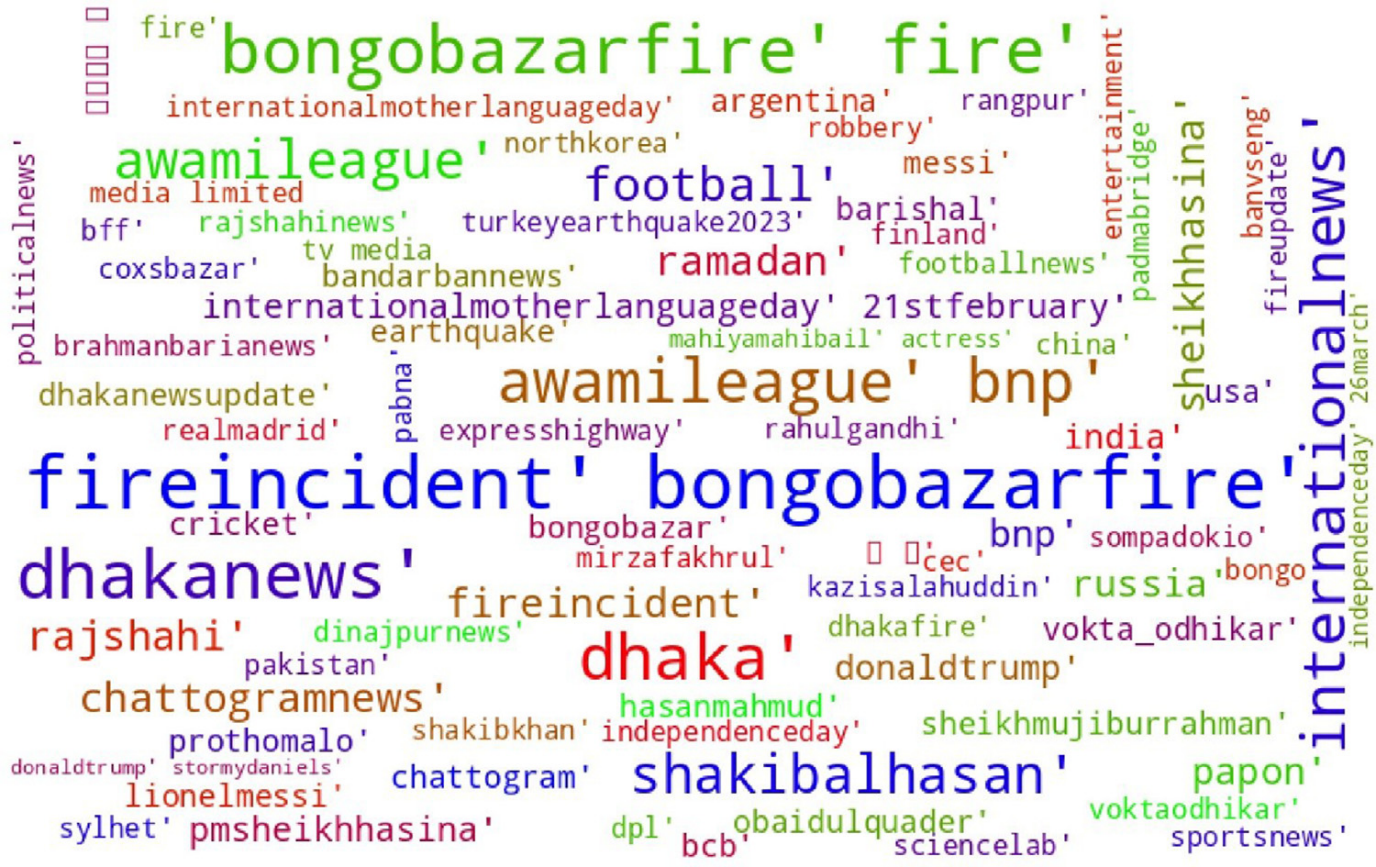
Word cloud for the hashtags of the news.

Some exploratory data analysis is carried out to comprehend the pattern of the data. In Fig. 2, a word cloud was plotted on the hashtags used in the data to identify the most frequently used words. In some news, people don't prefer showing their opinions. There are a total of 1,453 news articles found with no comments.

**Fig. 3:**
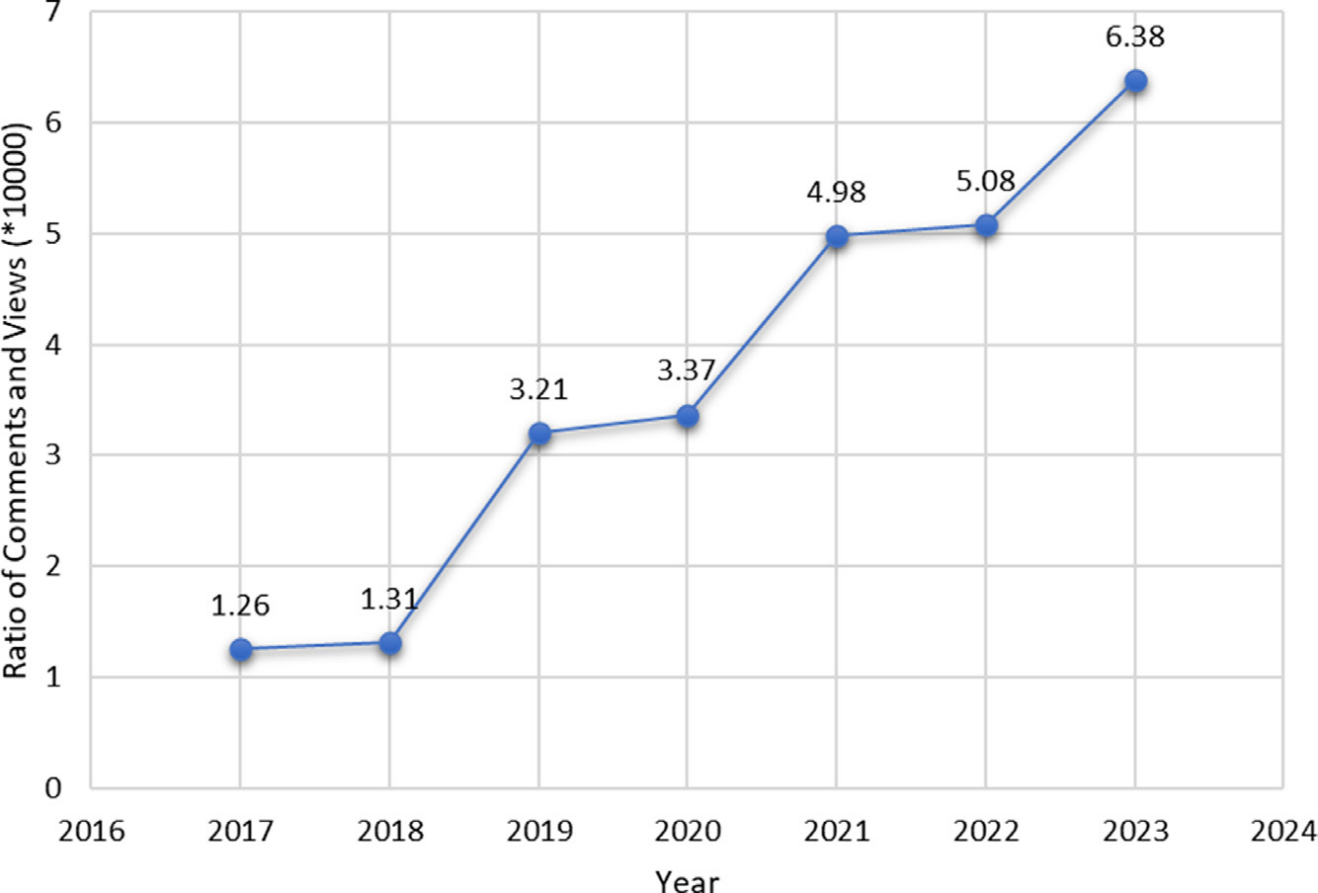
The ratio of comments and views for the data.

A notable change is also found in news views and comment proportion as the year goes along. Fig. 3 depicts the ratio of comments and views from the year 2017 to 2023. Some possible assumptions for the change could be that the number of users has increased over time, people have become more interactive on YouTube, people don't hesitate to express their opinions on public platforms, and many more.

**Fig. 4:**
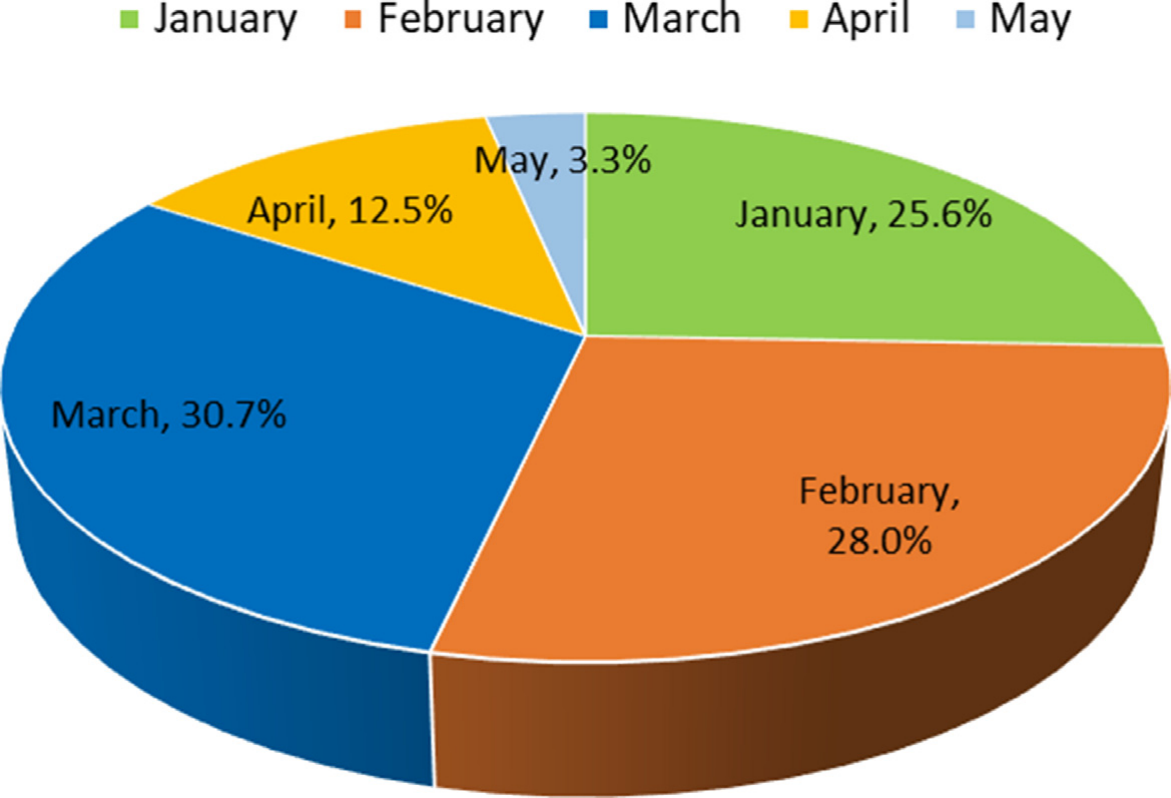
Rate of the news published in 2023.

According to Fig. 1, the year 2023 has the maximum number of samples in the data with 3,76,351 comments. To identify the time frame where the highest news was published, a pie chart is generated in Fig. 4. The diagram denotes that 30.7% of the news was published in March 2023. From January to March, the rate of publishing news was increasing. Whereas, the percentage declined during April.

**Fig. 5:**
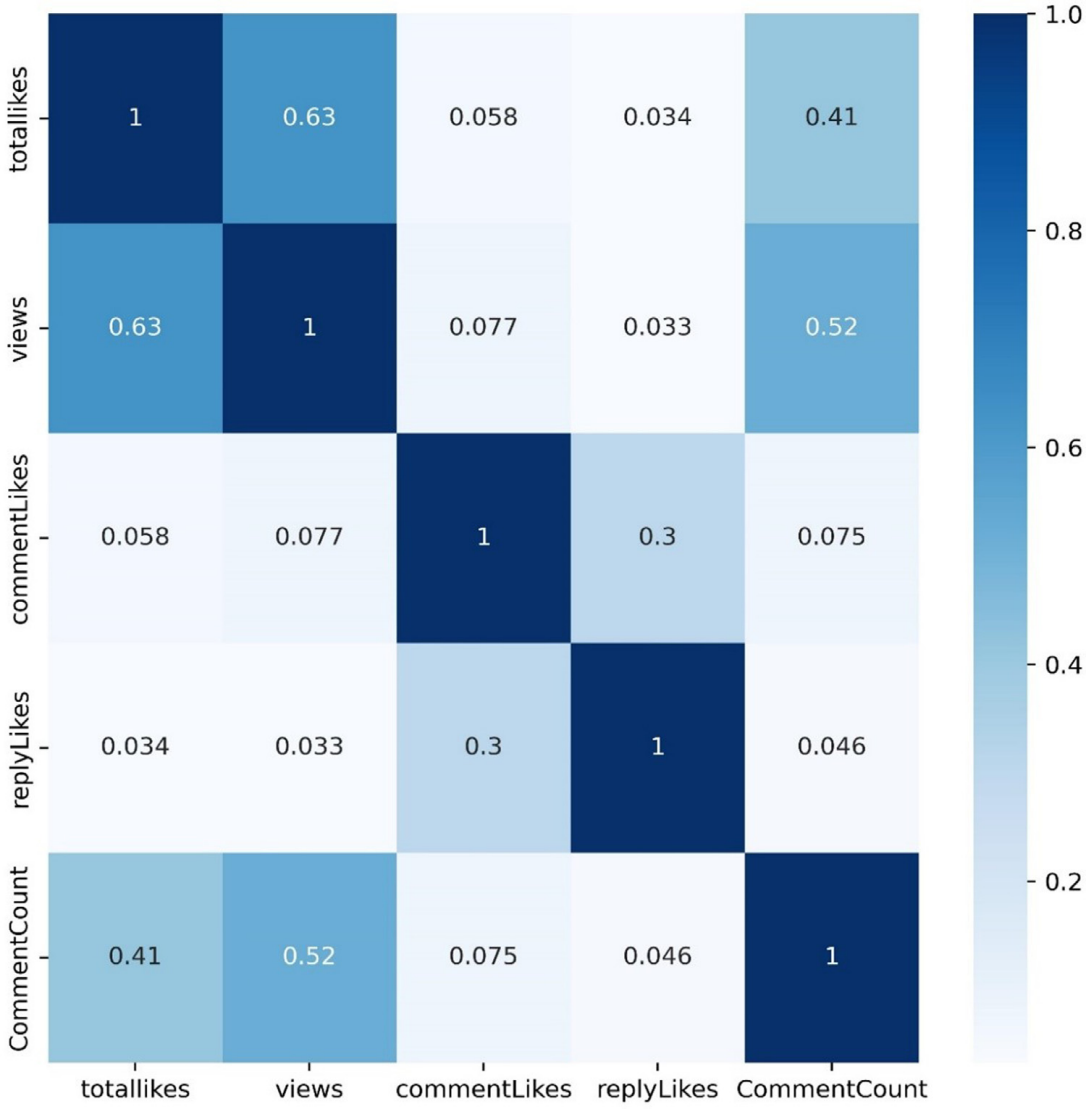
Correlation between the numerical attributes.

Fig. 5 displays the results of a correlation analysis that was carried out to determine the pattern of the data's numerical features. The correlation value depicts that all the attributes have a positive correlation among them. The maximum correlation is between the view of the videos and likes which is 0.63.

## Limitations

The data sentiments are not labeled. Manual data annotation may not be possible due to the volume of the data. There is a plan to implement semi-automated or automated sentiment polarization. The dataset is focused on a single YouTube news channel that publishes news in Bengali. The study in this context may include more valuable insights if multiple news channel data from different languages can be combined.

## Ethics Statement

Terms of Service – The dataset is completely anonymous and the data redistribution policies of the source platform have been complied with. The data is collected from a video streaming platform YouTube that grants permission to access their public content. Copyright – The original data belongs to the source of the data (SomoyTV) [1]. Privacy – The privacy of YouTube comments has been maintained by removing the name of the person. Scrapping policies – The data scrapping policies of YouTube have been maintained. No data for dislikes both for videos and comments were collected.

## CRediT authorship contribution statement

**Lomat Haider Chowdhury:** Methodology, Software, Data curation, Formal analysis, Visualization, Writing – original draft. **Salekul Islam:** Conceptualization, Formal analysis, Validation, Writing – review & editing. **Swakkhar Shatabda:** Formal analysis, Investigation, Validation, Writing – review & editing, Supervision.

## Data Availability

Bengali YouTube News Opinion Data from YouTube (Original data) (Mendeley Data).

## References

[1] W. Contributors, “Somoy TV - Wikipedia, the free encyclopedia”.

[2] Sunitha R. (2023). MABSA: a curated Malayalam aspect based sentiment analysis dataset on movie reviews. Data Brief.

[3] Adedeji Tolulope K., Oyero O., Aririguzoh S. (2019). Dataset on Online mass media engagements on YouTube for terrorism related discussions. Data Brief.

[4] Kumar Das R., Islam M., Akter Khushbu S. (2023). BTSD: A curated transformation of sentence dataset for text classification in Bangla language. Data in Brief.

[5] Hussein Ali A., Kumar H., Jack Soh P. (2021). Big data sentiment analysis of Twitter data. Mesopotamian J. BigData.

[6] Hande A., Priyadharshini R., Bharathi Chakravarthi R. (2020). Proceedings of the Third Workshop on Computational Modeling of People's Opinions, Personality, and Emotion's in Social Media.

[7] Hasan M., Islam L., Jahan I., Mannan Meem S., Rahman R.M (2023). Natural language processing and sentiment analysis on bangla social media comments on Russia–Ukraine war using transformers. Vietnam J. Comput. Sci..

[8] Atikur Rahman M., Kumar Dey E. (2018). Datasets for aspect-based sentiment analysis in bangla and its baseline evaluation. Data.

[9] Ahmed Masum M., Junayed Ahmed S., Tasnim A., Saiful Islam M. (2021). Proceedings of International Joint Conference on Advances in Computational Intelligence: IJCACI 2020.

[10] Haider Chowdhury L., Shatabda S., Islam S. (2023). Bengali YouTube news opinion data from YouTube. Mendeley Data.

